# DMfold: A Novel Method to Predict RNA Secondary Structure With Pseudoknots Based on Deep Learning and Improved Base Pair Maximization Principle

**DOI:** 10.3389/fgene.2019.00143

**Published:** 2019-03-04

**Authors:** Linyu Wang, Yuanning Liu, Xiaodan Zhong, Haiming Liu, Chao Lu, Cong Li, Hao Zhang

**Affiliations:** ^1^College of Computer Science and Technology, Jilin University, Changchun, China; ^2^Key Laboratory of Symbolic Computation and Knowledge Engineering, Ministry of Education, Jilin University, Changchun, China; ^3^Department of Pediatric Oncology, The First Hospital of Jilin University, Changchun, China

**Keywords:** RNA, secondary structure prediction, pseudoknot, deep learning, multi-sequence method, single-sequence method, improved base pair maximization principle

## Abstract

While predicting the secondary structure of RNA is vital for researching its function, determining RNA secondary structure is challenging, especially for that with pseudoknots. Typically, several excellent computational methods can be utilized to predict the secondary structure (with or without pseudoknots), but they have their own merits and demerits. These methods can be classified into two categories: the multi-sequence method and the single-sequence method. The main advantage of the multi-sequence method lies in its use of the auxiliary sequences to assist in predicting the secondary structure, but it can only successfully predict in the presence of multiple highly homologous sequences. The single-sequence method is associated with the major merit of easy operation (only need the target sequence to predict secondary structure), but its folding parameters are the common features of diversity RNA, which cannot describe the unique characteristics of RNA, thus potentially resulting in the low prediction accuracy in some RNA. In this paper, “DMfold,” a method based on the Deep Learning and Improved Base Pair Maximization Principle, is proposed to predict the secondary structure with pseudoknots, which fully absorbs the advantages and avoids some disadvantages of those two methods. Notably, DMfold could predict the secondary structure of RNA by learning similar RNA in the known structures, which uses the similar RNA sequences instead of the highly homogeneous sequences in the multi-sequence method, thereby reducing the requirement for auxiliary sequences. In DMfold, it only needs to input the target sequence to predict the secondary structure. Its folding parameters are fully extracted automatically by deep learning, which could avoid the lack of folding parameters in the single-sequence method. Experiments show that our method is not only simple to operate, but also improves the prediction accuracy compared to multiple excellent prediction methods. A repository containing our code can be found at https://github.com/linyuwangPHD/RNA-Secondary-Structure-Database.

## Introduction

RNA, the essential substance for all life, has played various roles in a variety of biological processes, such as translation (Kapranov et al., 2007), catalysis (Cech et al., 1981), and gene regulation (Storz and Gottesman, 2006). RNA contains a larger number of subunits that are called ribonucleotides, each of which is comprised of four possible bases: adenine(A), guanine(G), cytosine(C), or uracil(U). Under normal physiological conditions, these bases can bind with one another through the hydrogen-bond to form the secondary structure. Typically, the RNA secondary structure is a set of stems that are stacked with base pairs, while a base pair may be formed by three possible combinations of nucleotides, including A-U, G-C, and G-U, among which, A-U and G-C are called Watson-Crick pairs (Watson and Crick, 1953), while G-U is referred to as Wobble pair (Varani and Mcclain, 2000). The secondary structure information of RNA is of vital importance, since RNA functions largely depend on its secondary structure (Correll et al., 1997). Hence, predicting the RNA secondary structure is a bridge to understanding RNA functions. While the RNA secondary structure can be directly acquired through x-ray crystal diffraction or nuclear magnetic resonance; both of them are highly accurate and reliable, but they are restricted by their high price, slow and difficult operation. Therefore, it is necessary to develop mathematical and computational methods to predict the RNA secondary structure.

Computational methods have been used for over 40 years, dozens of methods have been proposed to predict the RNA secondary structure (with or without pseudoknots). These methods can mainly be classified into two categories based on the different prediction principles (Zhu et al., 2018): the multi-sequence method (MPM) (Hofacker and Stadler, 1999; Knudsen and Hein, 2003; Bernhart et al., 2008; Wilm et al., 2008) and the single-sequence method (SPM) (Eddy and Durbin, 1994; Zuker, 2003; Mathews, 2006; Zhu et al., 2018). The MPM can derive the secondary structure based on multiple homologous sequences using a comparative analysis model, which is the most accurate computational method for predicting the RNA secondary structure. However, it cannot predict the secondary structure when there are only some lowly homologous sequences, which can ascribe to its high requirement for homology sequences. The SPM can use a large number of parameters to predict the secondary structure, such as thermodynamic model (Zuker, 2003; Mathews, 2006) and statistical learning model (Eddy and Durbin, 1994; Zhu et al., 2018), and it can achieve favorably high accuracy of prediction results when those parameters are comprehensive and accurate. Unfortunately, the comprehensive and accurate parameters can hardly be obtained for different types of RNA through biological experiments or mathematical statistics, and the insufficient parameters may result in the low prediction accuracy in some RNA.

Pseudoknots have been shown in numerous studies to possess biological functions, which is thereby important to predict the secondary structure with pseudoknots. This paper aims to predict the RNA secondary structure with pseudoknots, which have been discovered in various RNA types, such as transfer-messenger RNA, ribosomal RNA, and viral RNA. Moreover, pseudoknots have been recognized to be involved in regulating translation, splicing, and ribosomal frame shifting (Brierley et al., 2007). Hence, predicting the RNA secondary structure with pseudoknots is closer to the natural structure, which contributes to a better understanding of RNA functions. To the best of our knowledge, very few tools have combined the merits of both MPM and SPM in predicting the RNA secondary structure with pseudoknots. Therefore, a new method is proposed in this paper to predict the RNA secondary structure with pseudoknots based on the Deep Learning and Improved Base Pair Maximization Principle (IBPMP), which is called “DMfold.” DMfold combines the advantages of both MPM and SPM while avoiding the disadvantages of them. For instance, similar to MPM, DMfold could use the known structure of RNA to help predict the secondary structure; meanwhile, unlike MPM, DMfold would use similar sequences instead of the highly homologous sequences, which reduces the requirement for auxiliary sequences and improve the algorithm availability. Similar to SPM, DMfold only needs to input the target sequence to predict the secondary structure, but it would use the deep learning model to automatically exact the RNA features that could avoid insufficient features and improve the prediction accuracy, which is different from SPM.

DMfold is a single model, which simultaneously using known structural data from multiple of families as learning and training data, to predict the secondary structure with pseudoknots of several different RNA. The secondary structure of RNA could be regarded to be composed of three types pseudoknot-free substructures (Danaee et al., 2018), each of which is represented by different types of symbols. Hence, the structural data of RNA can be transformed into dot-bracket sequences. Before the prediction process of DMfold, the structural data should be transformed into dot-bracket sequences (Danaee et al., 2018). Subsequently, the RNA sequences and dot-bracket sequences are used as the input and label in DMfold, respectively. After processing of RNA data, DMfold would use a deep learning model composed of encoder and decoder to complete the prediction from RNA sequences to dot-bracket sequences. Thereafter, DMfold would employ the IBPMP to obtain three pseudoknot-free substructures through selecting and combining the stems in the prediction dot-bracket sequences. Finally, the secondary structure with pseudoknots could be predicted by combining those substructures.

## Materials and Methods

### Data Collection and Processing

The original data used in this paper is same as the recent literature (Ward et al., 2017), which comes from the public database of Mathews lab. The dataset comprises 3,975 known RNA primary sequences and structure pairs. The sequences and structure pairs of 5sRNA, tRNA, tmRNA, and RNaseP are selected as the experimental data. Details of data usage can be found in Table S1. The following steps are employed to transform the raw data into mature data.

#### Data Cleaning

The tool of CD-HIT (The word_length is 10 and threshold is 1.0) (Fu et al., 2012) is adopted to remove the duplicate data and then coding to remove the data that contains the unknown bases.

#### Structure Format Conversion

**Figure 1:**
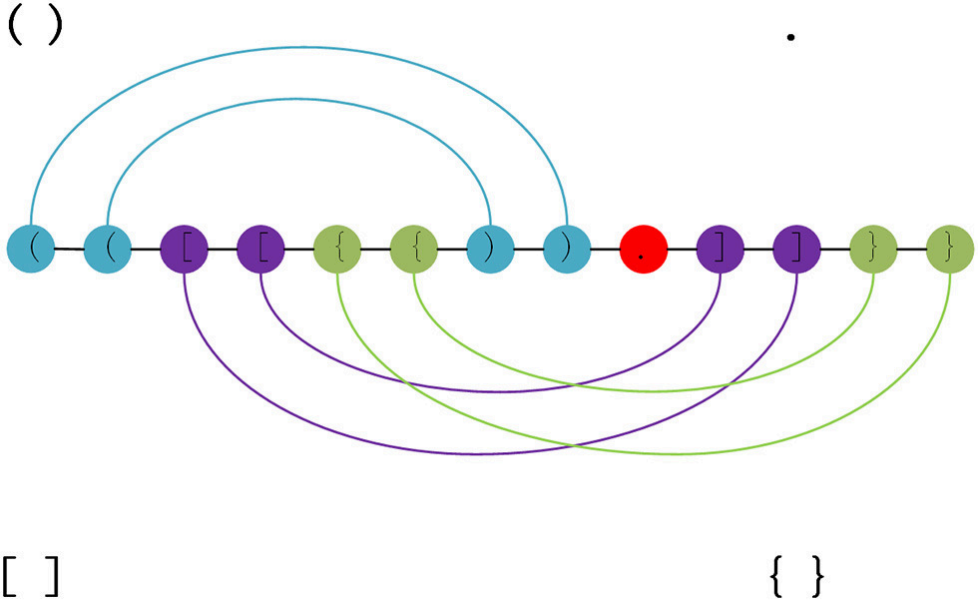
RNA structure can be decomposed into three pseudoknot-free substructures. Each color represents a substructure. There are three types of parentheses and a dot in the figure. The brackets represent the paired bases, the dots represent unpaired bases. Each pair of brackets corresponds to a separate substructure, and the edges, which represent the base pairs, are nested in a substructure.

As structural data is the non-sequence data, the original structure data format is transformed into the dot-bracket format (Danaee et al., 2018), which contains seven symbols. Figure 1 represents the transform regular between the original RNA secondary structure and dot-bracket sequences.

After these two steps, the clean RNA sequences and dot-bracket sequences could be obtained, which are used as the input and label in DMfold.

### Method

**Figure 2:**
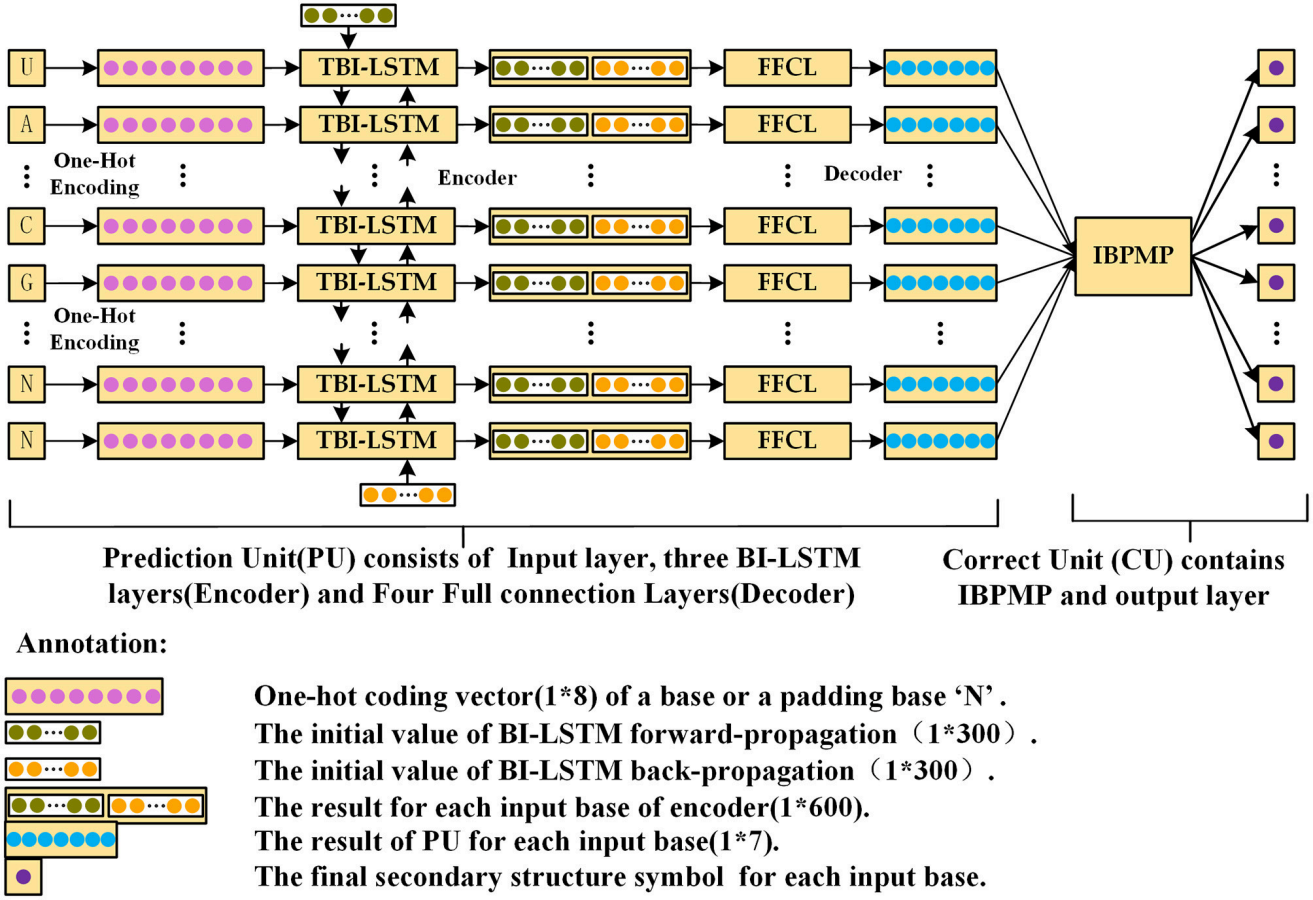
The schematic diagram of DMfold Architecture, which contains two parts: PU and CU. PU is a deep learning model, mainly responsible for predicting the input RNA sequences as dot-bracket sequences. CU is mainly to correct the prediction dot-bracket sequences and output the prediction secondary structure.

The Prediction Unit (PU) and Correction Unit (CU) are created as the two parts of DMfold. PU is a sequence to sequence Deep Learning model, which uses three-layer bidirectional LSTM (TBI-LSTM, which the dimension of the initial vector in each direction is 1^*^300) (Sutskever et al., 2014) as the encoder and four fully connected layer (FFCL) as the decoder to complete the prediction from RNA sequences to dot-bracket sequences. As there are some errors in the prediction results of PU, CU must be used to modify the prediction results and output the correct prediction dot-bracket sequence for each of RNA sequence. Specifically, CU could accomplish the task of modification and output the final prediction secondary structure based on IBPMP. Figure 2 displays the architecture of “DMfold.” As could be seen, DMfold first adopts the one-hot encoding to transform each of base into a vector (1^*^8). (Table 1 presents the rules of transformation between bases and one-hot vectors). Afterwards, DMfold could use those vectors (1^*^8) as the input of encoder, in which each vector (1^*^8) is encoded into a vector (1^*^600) containing the context information. Subsequently, the decoder could map the vector (1^*^600) to a secondary structure symbol, which employs the one-hot vector of the real symbol as the label (Table 2 shows the rules of transformation between dot-bracket symbols and one-hot vector). After all bases in an RNA sequence has been predicted by PU, the prediction results are then processed by CU and the prediction secondary structure with pseudoknots is output.

**Table 1:** The rules of transformation between bases and One-Hot vectors (Details can be found in Supplementary Materials).

**Input base**	**One-Hot encoding**
A	10000010
U	00101000
G	01000010
C	00100100
N (padding base)	00000000

**Table 2:** The rules of transformation between dot-brackets and One-Hot vectors (Details can be found in Supplementary Materials).

**Dot-bracket symbols**	**One-Hot encoding**
(	1000000
)	0000001
.	0001000
[	0100000
]	0000010
{	0010000
}	0000100
N (padding symbols)	0000000

#### Prediction Unit

PU is comprised of two parts: encoder and decoder, among which, the encoder is responsible for encoding the context-dependent bases into vectors (1^*^600) with context information, while the decoder is responsible for decoding those vectors (1^*^600) into the secondary structure symbols corresponding to those bases.

##### Encoder

The encoder model is built based on the LSTM architecture, which uses the memory cells to update and replace information, and is superior in finding and exploiting the long-range dependencies in context. Specifically, LSTM has been successfully applied in speech recognition (Graves et al., 2013), machine translation (Cho et al., 2014), and sequence to sequence learning (Sutskever et al., 2014). Figure S1 illustrates a single LSTM memory cell. As could be observed from the figure, some self-parameterized control gates are used to access, write and clear the cell. One advantage of using the gates to control information flow in the memory cell is that the gradient would be trapped in the cell, which could prevent from banishing too quickly, and it is a critical problem in the RNN model. The LSTM memory cell could be implemented as follows:

$$\begin{array}{l} i_{\dot{t}}=\;\sigma \left(w_{x_{i}}+\;w_{h_{i}}h_{t-1}+\;w_{c_{i}}C_{t-1}+\;b_{i}\right)\\ f_{t}=\;\sigma \left(w_{x_{f}}x_{t}+\;w_{hf}h_{t-1}+\;w_{Cf}C_{t-1}+\;b_{f}\right)\\ c_{t}=\;f_{t}C_{t-1}+\;i_{t}tanh\left(w_{xc}x_{t}+\;w_{hc}h_{t-1}+\;b_{c}\right)\\ O_{t}=\;\sigma \left(w_{xo}x_{t}+\;w_{ho}h_{t-1}+\;w_{co}C_{t}\;+\;b_{o}\right)\\ h_{t}=\;O_{t}tanh\left(C_{t}\right) \end{array}$$

where σ is the logistic sigmoid function, while i, f, o, and c are the input gate, forget gate, output gate and cell vector, respectively, and all of them are at the same dimension as the hidden vector h (The dimension is 1^*^300). Meanwhile, w denotes the weight matrices and the b indicates the bias vectors.

In this paper, the RNA sequences are considered as long-distance context-dependent sequences. Hence, it is necessary to access both past and future features for each base in the task of predicting from the RNA sequences to the dot-bracket sequences. In DMfold, a three-layer BI-LSTM model is used as the encoder, which consisted of both forward and backward networks. The forward LSTM processed the RNA sequences from left to right, whereas the backward LSTM processed in the reverse order. Therefore, two hidden state sequences could be obtained, one from the forward network $\left(\vec{h_{1}},\;\vec{h_{2}},\;\ldots ,\;\vec{h_{n}}\right)$, and the other one from the backward one $\left(\overset{⃖}{h_{1}}\;,\;\overset{⃖}{h_{2}},\;\ldots ,\;\overset{⃖}{h_{n}}\right)$. Moreover, the encoder could concatenate the forward and the backward hidden state of each input vector, resulting in $h_{m}=\left[\;\vec{h_{m}\;};\;\overset{⃖}{h_{m}}\right]$. In this way, the encoding vector (1^*^600) of each input vector (1^*^8) could be obtained. Figure S2 is a schematic diagram of the encoder. After inputting a vector (1^*^8), the feature of multi-layer BI-LSTM could be used to encode the input vector (1^*^8) with its context information into a vector (1^*^600).

##### Decoder

It is necessary to map the vector (1^*^600) to an RNA secondary structure symbol after a base is encoded to a vector (1^*^600). In this paper, a four-layer fully connected neural network is proposed to accomplish the mapping work. Figure S3 shows the architecture of the decoder, consisting of one input layer, two hidden layers, and one output layer. The numbers of nodes in each layer are 600, 1024, 512, and 7, respectively. In the network, ReLU is used as the activation function, while the vector (1^*^600) is used as the input. The fully connected neural network could be implemented as follows:

y=ReLU(wx+b)

where ReLU is the activation function, w is the weight matrices, b is the bias vectors. x and y are the input and output between any two layers.

There are seven nodes in the output layer, each of the node contains an output value, and the largest value is set as 1, whereas the other nodes are set as 0, which correspond to the one-hot vector of dot-bracket symbols. Hence, the result of each base is 1000000, 0000001, 0001000, 0100000, 0000010, 0010000, or 0000100.

##### PU training and testing

The clean data is divided into three sub-sets: including (1) pure testing set containing 10% of all the clean data that is untouched during the learning phase; and (2) the training set and validation set, which are created by the 10-fold cross-validation for the remaining clean data. As the RNA sequences and Dot-Bracket sequences vary in length, and they should be intercepted or padded to have the same length. For those sequences with the length of <300, “N” is padded to those sequences until the length is equal to 300. Meanwhile, the remaining sequences are intercepted from the beginning into multi sub-sequences that contain 300 bases. The overlap length between two consecutive sub-sequences is 200. Finally, “N” is padded to the sub-sequences with the length of <300, and the same length sequences are used to train and test PU.

In PU, the cross-entropy loss (CEL) is employed to quantify the training errors and the goal is to minimize CEL. The complete training details are given below:

1. A normal distribution with a standard deviation of 0.1 initial all weights and biases is used.
2. The dropout (the value is 0.9 in the encoder and decoder, respectively) function is used to prevent overfitting.
3. The Back-Propagation through Time (BPTT) algorithm is employed to compute the gradient, while the Adaptive Moment Estimation Optimizer algorithm is utilized to reduce the gradient.
4. Subsequently, PU is trained for a total of 50 epochs at a learning rate of 0.002. After each epoch, the learning rate decay is used to reduce the learning rate.

**Figure 3:**
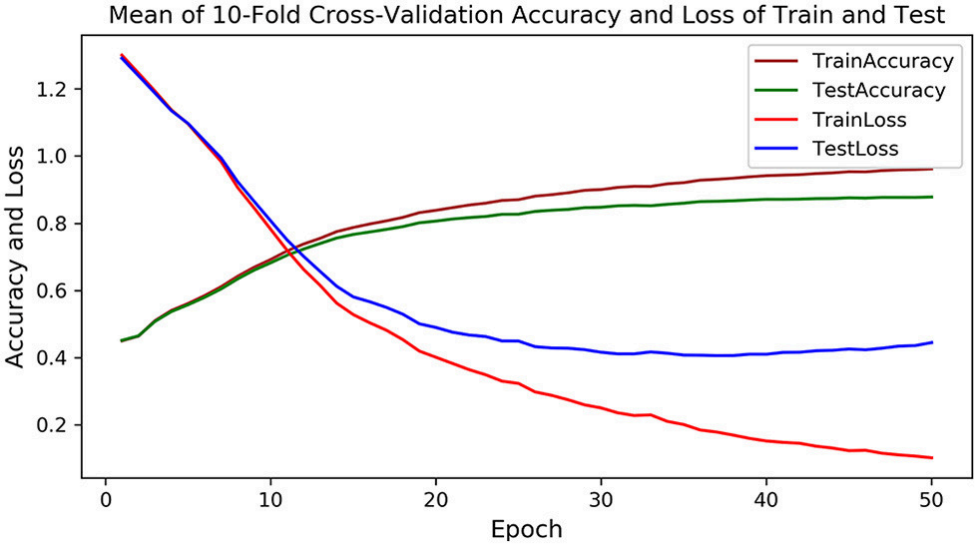
The mean accuracy and loss of training and testing in the 10-fold cross-validation experiments, in which the brown and green curve represents the accuracy of training and testing, and the red and blue curve represents the loss of training and testing.

As PU is a deep learning model, the 10-fold cross-validation is employed to verify the stable performance of PU. In each fold experiment, 50 epochs are trained, and the loss and accuracy of each epoch are recorded for both the training set and the testing set. Figure 3 shows the average loss and accuracy at each epoch in the 10-fold cross-validation experiments. As could be observed, after the 40th epoch, the test loss and accuracy are tending to be stable, with the highest testing accuracy of 87.8%, indicating that PU could successfully complete the prediction from RNA sequences to dot-bracket sequences.

#### Correction Unit

After an RNA sequence has been predicted by PU, those prediction results of the padding bases are removed and the multiple sub-sequences prediction results are spliced. The processed prediction results are the input of CU. Typically, the RNA secondary structure could be considered as a combination of stems and loops (Sakakibara et al., 2007), each stem contains two complementary regions: 5′ complementary region (5′-CR) and 3′ complementary region (3′-CR), and each loop is comprised of multiple unpaired bases. Hence, according to different substructure, the continuous “1000000,” “0100000,” or “0010000” represent the prediction 5′-CR (5′-PCR) in the prediction results; whereas the continuous “0000001,” “0000010,” or “0000100” stand for the prediction 3′-CR (3′-PCR) in the prediction results, and the continuous “0001000” indicates the prediction loop in the prediction results.

According to different types of pseudoknot-free substructures, PCRs could be divided into three sets, each set contains all the 5′-PCRs and 3′-PCRs of a pseudoknot-free substructure, such as all continuous “1000000” and “0000001” in a set, all continuous “0100000” and “0000010” in a set, and all continuous “0010000” and “0000100” in a set. In CU, the IBPMP is employed to find the optimal compatible stem combinations for each set, which represent the prediction pseudoknot-free substructures. Then, those three pseudoknot-free substructures are combined to obtain the prediction secondary structure with pseudoknots.

##### Some definitions and operation

###### Matrix:

An n×*n* upper triangular matrix is created to store all the potential maximal stems for each RNA sequence (n represents the sequence length), in which the specific row and column stand for the corresponding nucleotides in the sequence. For example, row *i* and column *j* represents the *ith* and *jth* nucleotides, respectively. Accordingly, a position in the matrix that could form a base pair would then be marked as 1; otherwise, it would be denoted as 0. Each of the maximal stem represents a diagonal line in the Matrix.

###### Stem:

For an RNA sequence, if two disjoint areas could be paired reversely to form m continuous base pairs, then those m continuous base pairs are deemed as a stem. A stem could be expressed as a triplet *stm* = (*S, E, L*), among which, S and E are the subscripts that are the closest to the 5′ end and 3′ end, respectively, whereas L represents the length.

###### Compatible:

For any two stems in the RNA sequence *stm*_1_ = (*S*_1_, *E*_1_, *L*_1_) and *stm*_2_ = (*S*_2_, *E*_2_, *L*_2_), if (*E*_1_ < *S*_2_) or (*E*_2_ < *S*_1_) or (*S*_1_+*L*_1_−1 < *S*_2_ and *E*_2_−*L*_2_+1 < *E*_1_ ) or (*S*_2_+*L*_2_−1 < *S*_1_ and *E*_1_−*L*_1_+1 < *E*_2_), then stem_1_ is compatible with stem_2_; otherwise, those two stems are incompatible.

###### Rate:

After multiple (>1) PCRs have been performed to search for the optimal combination of stems, the usage rate of those PCRs should be calculated according to the stems. Firstly, those PCRs should be divided into two categories, including 5′-PCR and 3′-PCR, with the number bases of H and G, respectively. For the combination stems, the number of bases in 5′-CR and 3′-CR which contained in 5′-PCR and 3′-PCR are counted as h and g, respectively. Besides, the usage rates of 5′-PCR and 3′-PCR are calculated according to the following formulas: 5′-Rate = h/H and 3′-Rate = g/G, respectively, while that of all PCRs is recorded as Rate = (5′-Rate + 3′-Rate)/2.

###### Extend:

When extending the stems in a combination, those stems are first located to the corresponding positions of Matrix before they are extended using the maximal stems. The extension parts of each stem would not overlap with other stems; meanwhile, those extension parts of any two stems would not overlap.

##### IBPMP

**Figure 4:**
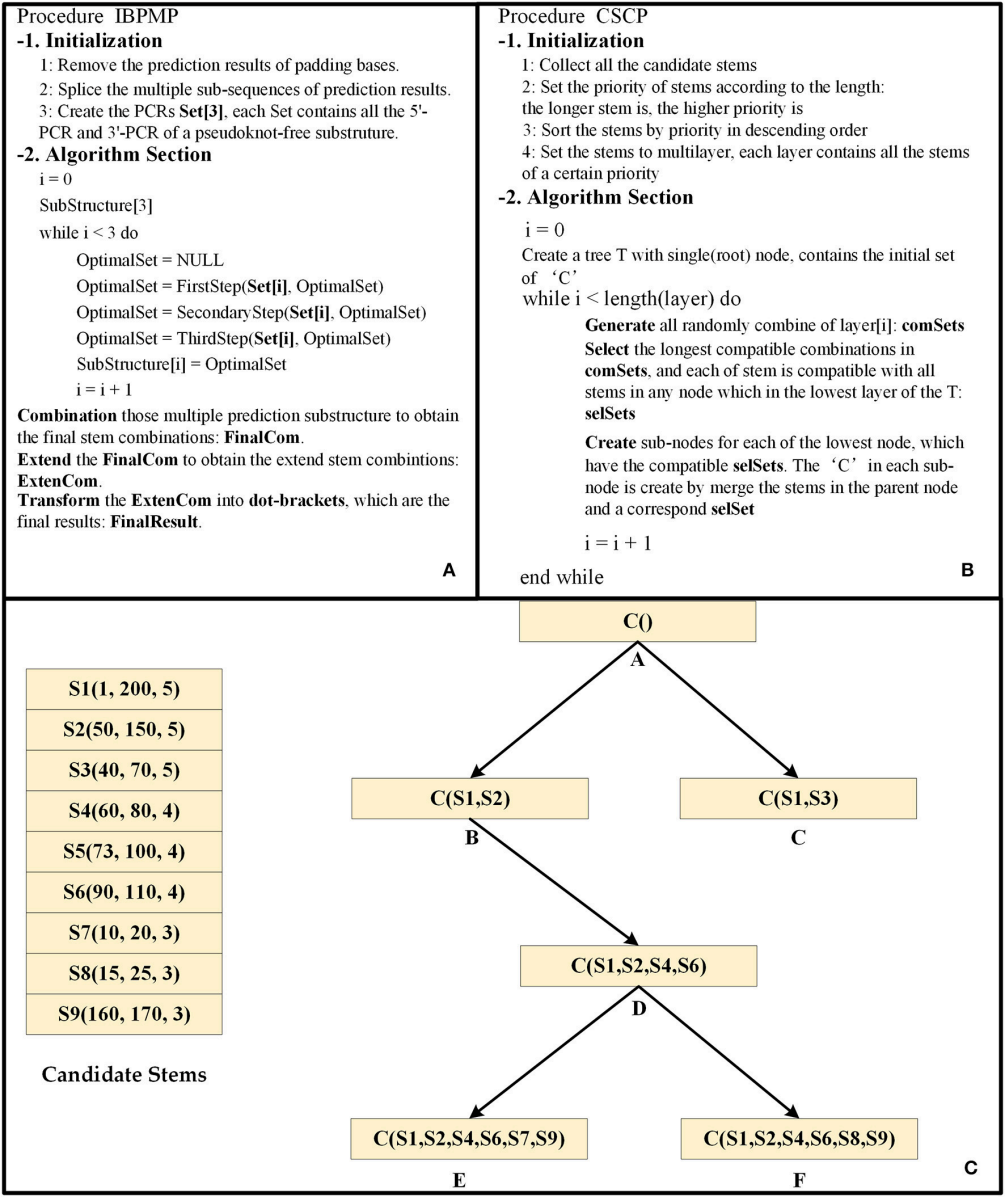
The principle diagram of IBPMP. **(A)** is the procedure of IBPMP, which contains two parts: initialization and algorithm section. In the initialization, the procedure processes the prediction results of PU as the input of CU. In the algorithm section, it obtains the prediction secondary with pseudoknots. See below for details of FirstStep, SecondaryStep, and ThirdStep. **(B)** The procedure of CSCP, which contains two parts: initialization and algorithm section. In the initialization, it collects all stems and set priority for them. In the algorithm section, it obtains the optimal stem combinations. **(C)** is an example of the CSCP.

For each of the PCRs set, the original Base Pair Maximization Principle (Eddy, 2004) would be used to search for the longest combination of stems if all PCRs are completely correct. Unfortunately, there are always some errors in the set, so the IBPMP instead of the original principle is used to find the optimal compatible stem combinations, which might not the longest stem combination. The difference between IBPMP and original principle could mainly be reflected in two aspects. On the one hand, different from the original principle by which stems could be formed in all complementary regions (Eddy, 2004), the new principle stipulates that stems only could be produced between 5′-PCR and 3′-PCR (the relationships between the 5′-PCR subscripts i and j, and the 3′-PCR subscripts p and q follow the order of i<j<p<q). On the other hand, unlike the original principle in which all the stems are selected in the RNA sequence simultaneously as the candidate stems and each stem has the same priority to find the longest compatible stem combination (Eddy, 2004), the new principle selects the candidate stems in multiple steps, and different priorities are used in each step to combine the candidate stems. Noteworthily, the time and space complexities of IBPMP are greatly reduced compared with the original principle. Figure 4A is the procedure of IBPMP.

The candidate stems combination principle (CSCP) is the key of IBPMP, which stipulates that a longer stem is associated with a higher priority, and those stems would be combined based on the priority of candidate stems from high to low (Those stems with the same priority would be combined simultaneously). Figures 4B,C shows the principle of CSCP, which Figure 4B is the procedure of CSCP and Figure 4C is an example. As could be observed in Figure 4C, each node contains a “C” set, which represents the optimal combination of the node. Of them, the “C” in the root node stand for the initial set (might be empty), while the other “C” are generated layer-by-layer by adding several new stems to the “C” in their parent nodes, the new stems are not only compatible between themselves but also compatible with all stems in their parent nodes. In addition, the “C” contained in the lowest layer leaf nodes stand for the optimal compatible combination results. When all the candidate stems of the same priority are combined, all the random combinations of those stems would be generated first, with the number ranging from 1 to O (O represents the number of those stems). Later, the compatible longest stem combinations would be selected, and each of stem is compatible with all the stems in their father nodes. Finally, all the selected combinations are added to “C” of their compatible father nodes, respectively, and different sub-nodes would then be generated with new “C.” Such as (shown in Figure 4C), seven stem combinations, including (S4), (S5), (S6), (S4, S5), (S4, S6), (S5, S6), and (S4, S5, S6), would first be produced when S4, S5, and S6 are combined. Then, those compatible longest stem combinations would be selected, and each of stem is compatible with all stems in B or C. Finally, (S4, S6) is selected as the new stem combination, and S4 and S6 are later added to “C” in node B to generate node D.

There are two kinds of candidate stem selection and combination in each step, among which, the first kind of selection is to select all the stems among multiple PCRs as the candidate stems to produce the first kind of stem combinations based on CSCP. If the first kind of stem combinations could satisfy the conditions of the current step, then collect all the appropriate stems in those combinations (The secondary kind of selection) as the candidate stems. Afterwards, all the secondary selection stems would be used to produce the secondary kind of stem combinations based on CSCP, which stand for the optimal results of each step. The details of each step are presented below:

The first step: Firstly, all PCRs are randomly combined, and each of the combinable result involves n (*n* ≥ 2) PCRs. Afterwards, results containing the PCRs that could not form the stems would be removed; for instance, the minimum subscript PCR is 3′-PCR or the maximum subscript PCR is 5′-PCR. For each of the remaining combinable result, the bases of 5′-PCRs and 3′-PCRs in the rows and columns of Matrix are located, respectively. All the stems in all fixed areas are collected (The first kind of collection) as the candidate stems, and the subscripts of 5′-PCR i and j as well as those of 3′-PCR p and q follow the order of i<j<p<q. For example, if some bases contained in a 5′-PCR have the subscripts from ith to jth, and some contained in 3′-PCR have the subscripts from pth to qth, then the ith to jth rows and pth to qth columns in Matrix would be located, and all the stems of the area would be collected in the case of i<j<p<q. Based on the collected candidate stems, CSCP (the initial “C” is empty) is used to obtain the first kind of optimal stem combinations and to compute the rate. If the rate is 1, then all the appropriate stems would be collected (The secondary kind collection). After processing all the remaining combinable results, all the collected stems are the candidate stems of the step. Secondly, the CSCP (the initial “C” is “OptimalSet,” which is an empty set before the first step) is also employed to obtain the secondary kind of optimal stem combinations “OptimalSet.” Finally, the PCRs that have formed stems would be removed. The above operation would be repeated, the initial value of n is 2, and each of epoch operation is increased by 1 until the value of n approaches to 4.

The secondary step: Firstly, all the remaining PCRs are randomly combined, and each of the combinable result contains two PCRs. Then, the results containing PCRs that could not form stems would be removed. For each of the remaining combinable result, the bases of 5′-PCRs and 3′-PCRs would be located in the rows and columns of Matrix, respectively. All the stems in the fixed areas are then collected (The first kind of collection) as the candidate stems, and the subscripts of 5′-PCR i and j as well as those of 3′-PCR p and q follow the order of i<j<p<q. Based on the collected candidate stems, CSCP (the initial “C” is empty) is used to obtain the first kind of optimal stem combinations and to compute the rate. If one single usage rate (5′-Rate or 3′-Rate) is 1, then all the appropriate stems would be collected (The secondary kind of collection). After processing all the remaining combinable results, all the collected stems represent the candidate stems of the step. Secondly, the CSCP (the initial “C” is “OptimalSet”) is also utilized to obtain the secondary kind of optimal stem combinations “OptimalSet.” Finally, the PCRs with the single usage rate of 1 would be removed.

The third step: In this step, all the remaining PCRs are randomly combined first of all, and each of the combinable result contains two PCRs. Then, the results containing PCRs that could not form stems would be removed. For each of the remaining combinable result, the bases of 5′-PCRs and 3′-PCRs would be located in the rows and columns of Matrix, respectively. Then, all the stems in the fixed areas would be collected (The first kind collection) as the candidate stems, and the subscripts of 5′-PCR i and j as well as those of 3′-PCR p and q follow the order of i<j<p<q. Subsequently, the CSCP (the initial “C” is empty) is employed to obtain the first kind of optimal stems combination and to compute the rate. All the appropriate stems would be collected (The secondary kind of collection) as the candidate stems if the usage rate is >0.6. After processing all the remaining combinable results, all the collected stems are the candidate stems of the step. Secondly, the CSCP (the initial “C” is “OptimalSet”) is then used to obtain the secondary kind of optimal stems combination “OptimalSet.”

Repeating the above operation allows to obtain the prediction pseudoknot-free substructures of each PCRs set, and those substructures are then randomly combined to produce the final stem combinations, each of which only contains one substructure of each PCRs set. Additionally, the final stem combinations would also be extended to get the prediction secondary structure with pseudoknots. Eventually, the prediction structure would be transformed into the dot-bracket sequences and output them.

#### Performance Measurement

For the same RNA, the prediction structures of some methods contain pseudoknots and some don't contain pseudoknots. Since the pseudoknots are formed by the intersection of stems and the non-pseudoknot structures are formed by nested stems. Therefore, we can calculate the accuracy of the base pairs to represent the accuracy of prediction structures. So that, the prediction structures can be compared between different methods.

To estimate the accuracy of the prediction results for DMfold and other methods, the indexes of sensitivity (SEN) and positive predictive value (PPV) are commonly used (Seetin and Mathews, 2012), among which, SEN could measure the ability to find the positive base pairs, while PPV could measure the ability of not folding false positive base pairs. To be specific, SEN and PPV could be defined by equation (1) and (2), respectively.

(1) SEN=TP/(TP+FN)

(2) PPV=TP/(TP+FP)

where TP (true positive) is the number of matched bases that are correctly predicted, FN (false negative) is the number of existing matched bases that are not predicted, and FP (false positive) is the number of matched bases that are incorrectly predicted.

Generally, the requirements of SEN and PPV could not be satisfied simultaneously when comparing the accuracy of those prediction results. Therefore, the F-score (Yonemoto et al., 2015) is used to comprehensively evaluate the prediction results, which is harmonic mean of SEN and PPV. Specifically, the value of F-score [can be defined by equation (3)] ranges from 0 to 1, 0 indicates that the prediction structure has no common base pair with the real structure, whereas 1 suggests that the prediction structure is the same to the real structure.

(3) F−score =  2 ∗ ((SEN + PPV) / (SEN ∗ PPV))

## Results

In this section, the prediction results of our method would be presented and our method would be compared with several excellent methods, including mfold (Zuker, 2003), RNAfold (Zuker and Stiegler, 1981), cofold (Proctor and Meyer, 2013), Ipknot (Kengo et al., 2011), and Probknot (Bellaousov and Mathews, 2010). Among those methods, mfold, RNAfold, and cofold could predict the pseudoknot-free secondary structure, while Ipknot and Probknot could predict the secondary structure with pseudoknots. Therefore, those comparison methods include methods for predicting pseudoknots and pseudoknots-free, which can compare the performance of our method more comprehensively. In this paper, the prediction results of multiple methods are compared in two aspects: performance and structure visualization. The performance comparison is mainly to test the accuracy of predicting base pairs, while the structure visualization comparison is mainly to testing which results is closer to the natural structure. To facilitate comparison among methods, the structure with the highest value of F-score would be selected as the prediction structure when an RNA sequence is predicted by a method.

### Performance Comparison

In this paper, data in the Testing set is classified according to the RNA family, and the prediction results are compared among different families. When calculating the parameters (SEN, PPV, and F-score) of DMfold, the values of those parameters in each fold experiment are obtained, which represent the means of all the RNA sequences in different families. Accordingly, the means of those parameters in 10-fold cross-experiments stand for the prediction parameters of DMfold. When calculating the parameters (SEN, PPV, and F-score) of other methods, all parameters of each RNA sequences would be obtained, and the mean parameters in different families represent the prediction parameters of other methods.

Table 3 compares the prediction results of our method and other methods on tRNA and 5sRNA, which represent of the short RNA sequences with the length of 70–200. It could be obviously seen that the SEN, PPV, and F-score of DMfold are higher than those of the other methods in terms of tRNA and 5sRNA. Therefore, DMfold is superior to other excellent methods for short RNA sequences. Table 4 compares the prediction results of our method and other methods on tmRNA and RNaseP, which represent the long RNA sequences that are 300–500 in length. It could be discovered that the parameters of SEN, PPV, and F-score of DMfold are higher than those of other methods in tmRNA. SEN and F-score in RNaseP of DMfold are at the common level, but PPV is optimal, suggesting that the prediction results of DMfold in RNaseP are associated with the least proportion of false positive bases. These two tables have verified that DMfold could effectively predict the secondary structure of both short and long RNA sequences.

**Table 3:** The comparison between DMfold and other methods on 5sRNA and tRNA.

**Method**	**tRNA**			**5sRNA**		
	**SEN**	**PPV**	**F-score**	**SEN**	**PPV**	**F-score**
mfold	0.741	0.708	0.722	0.708	0.675	0.690
RNAfold	0.708	0.634	0.667	0.613	0.550	0.579
Cofold	0.627	0.595	0.609	0.578	0.548	0.562
IPknot	0.787	0.775	0.774	0.485	0.555	0.512
Probknot	0.745	0.635	0.683	0.562	0.538	0.548
DMfold	**0.934**	**0.946**	**0.937**	**0.928**	**0.930**	**0.927**

*The bold value is the maximum of each column*.

**Table 4:** The comparison between DMfold and other methods on tmRNA and RnaseP.

**Method**	**tmRNA**			**RNaseP**		
	**SEN**	**PPV**	**F-score**	**SEN**	**PPV**	**F-score**
mfold	0.558	0.518	0.536	**0.656**	0.605	**0.624**
RNAfold	0.470	0.433	0.448	0.564	0.499	0.526
Cofold	0.358	0.329	0.342	0.518	0.481	0.495
IPknot	0.463	0.495	0.476	0.587	0.640	0.604
Probknot	0.457	0.410	0.431	0.583	0.531	0.551
DMfold	**0.630**	**0.830**	**0.706**	0.547	**0.728**	0.619

*The bold value is the maximum of each column*.

### Structure Visualization Comparison

**Figure 5:**
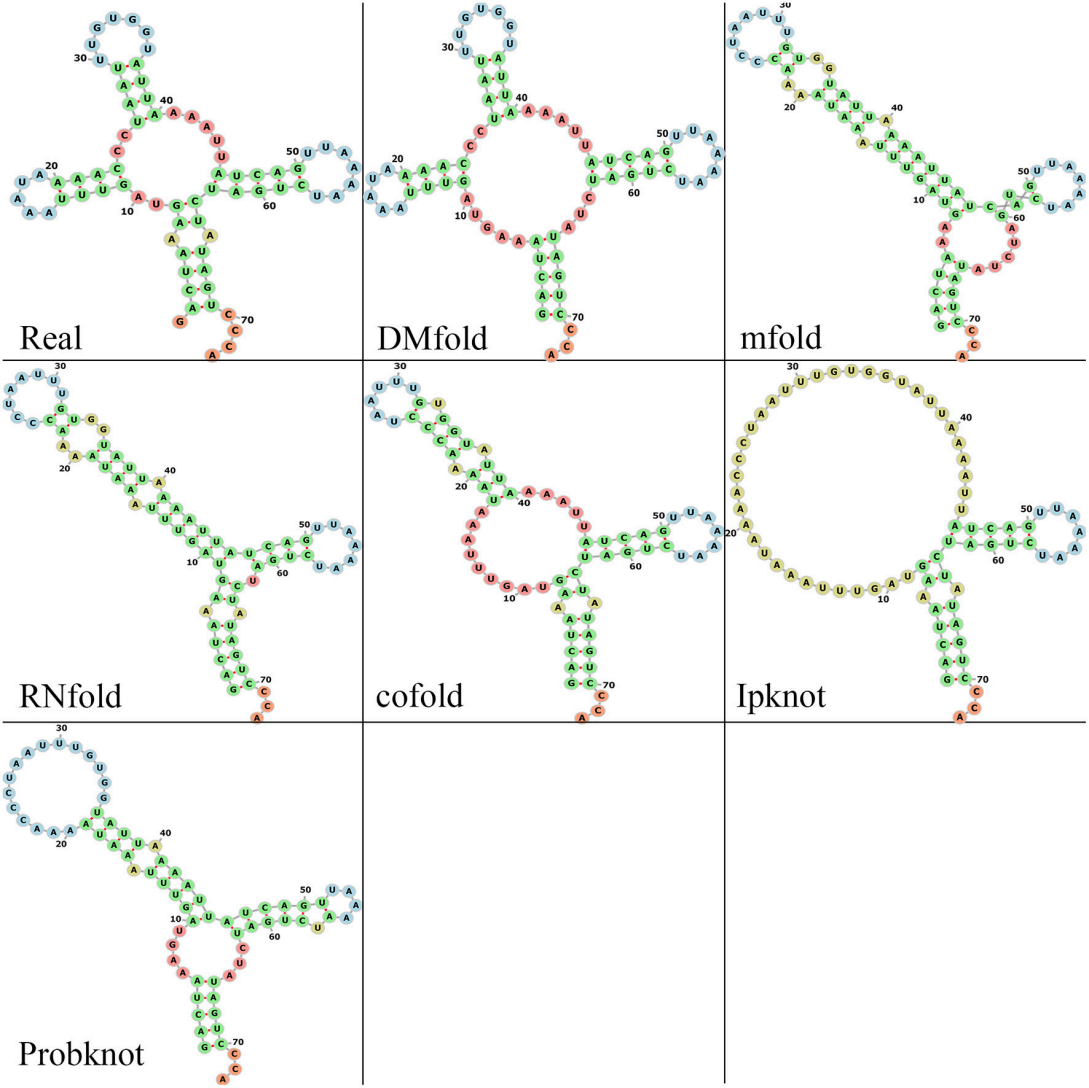
The visualization results of multiple methods and real structure. Green bases represent the stem. Red bases represent the bifurcation loop and unpaired single chain. Blue bases represent the hairpin loop. Yellow bases represent the interior and bulge loop.

Since the function of RNA is highly correlated with the shape of its secondary structure, we compare different methods by observing the visualization maps. First, a tRNA molecule (tRNA_tdbR00000143-Asterias_amurensis-7602-His-QUG) is randomly selected in the testing set, and the prediction results of those six methods are obtained. Then use the forna tool (Gruber et al., 2015) to get the visualization maps of those prediction results. Figure 5 shows the visual representation of the real and prediction structures. As shown, the DMfold structure and the real structure have four branches on the bifurcation loop, forming the typical clover shape of tRNA, which is the key to transport amino acids. The prediction structures of mfold, RNAfold, cofold, and ProbKnot lack a branch in bifurcation loop, which can seriously affect the function of tRNA. The IPknot method only successfully prediction two branches, which is also seriously inconsistent with the real structure. Although the structure predicted by our method is not completely correct, it is the closest to the natural structure compared to other methods. Therefore, our method is more conducive to the study of RNA function. See the Supplementary Materials for the visualization comparison of the other three families (Figures S4–S6).

## Discussion

In this paper, a new method is proposed to solve the problem of predicting the RNA secondary structure with pseudoknots. Actually, computational methods have been used for over 40 years to predict the secondary structure of RNA. Although many prediction methods have been proposed, only a few of them can predict the RNA secondary structure with pseudoknots, since it is an NP-hard problem (Rivas and Eddy, 1999). In the traditional computational methods, the prediction of pseudoknots will greatly add to the algorithmic complexity. Hence, many methods would not predict pseudoknots or would only predict some common pseudoknots for the sake of reducing the algorithmic complexity (Rivas and Eddy, 1999). Different from the traditional computational methods, our method transforms the pseudoknots problems into the pseudoknots-free problems, which could predict the RNA secondary structure with all kinds of pseudoknots in a reasonable complexity. More importantly, it can be found in the results section, our prediction results are closer to the natural structure. Hence, our method is more beneficial to study the function of RNA.

The novel of our method is that first combines the Deep learning and IBPMP to solve the problem of predicting the secondary structure with pseudoknots. Unlike the traditional computational methods using MPM or SPM to predict the secondary structure, our method has taken full use of the advantages of those two main methods. Our method uses the auxiliary sequence to help predict RNA secondary structure and uses the Deep Learning model to automatically extract RNA features without using the energy or statistical parameters in the traditional computational methods. Compared with MSM, only the target sequence is needed in our method as the input, which has greatly simplified the method operation. Besides, compared with SPM, our method could effectively break through the restriction of parameter insufficiency in traditional computational methods.

Moreover, in order to improve the credibility of our method, the 10-fold cross-validation experiments are employed to train and test our method, and both short and long RNA are included in the experimental data. As could be discovered from the results section, the prediction accuracy of our method in short RNA sequences is greatly improved relative to that of the other methods. In long RNA sequences, the accuracy of our method is not as good as that in short RNA sequences, but the prediction results are also improved. Two reasons may be responsible for such phenomenon; on the one hand, the topology of short RNA sequences is simple and existing data can support short sequences learning and predicting; on the other hand, the topology of long RNA sequences is complex and the existing long sequences are insufficient to support the learning and predicting. These results indicate that the accuracy of our method on long RNA sequences remains to be further improved with the accumulation of known structural data.

The improved prediction accuracy can be due to that different RNA in the different microenvironment. Hence, these differences in microenvironment may result in RNA folding along different rules, indicating that the traditional computational methods taking the common folding rules are not favorable for predicting the multi-type RNA secondary structures, especially those with pseudoknots. On this basis, our method learning from different types of RNA and predicting the similar RNA structure, which could effectively avoid the low prediction accuracy caused by single rules. Compared with the traditional computational methods, our method is more suitable for predicting the multiple different types of RNA secondary structure.

Our method is associate with many advantages, nonetheless, it is also inevitably link with certain limitations. Because our method contains a deep learning model, it needs a large number of similar RNA with known structures to learn features for different types of RNA. Therefore, the prediction accuracy might be reduced in the presence of insufficient similar sequences, so the use space of our method is partly limited. Despite some limitations in the use space of our method, the use space is promising to be gradually growing along with the increase in the number of secondary structures found.

## Author Contributions

YL conceived and directed the project. LW designed the study, wrote the manuscript. HL and XZ revised the manuscript critically for important intellectual content. CoL and ChL collected the data and coded the procedure. HZ reviewed the data. All authors have read and approved the final manuscript for publication.

### Conflict of Interest Statement

The authors declare that the research was conducted in the absence of any commercial or financial relationships that could be construed as a potential conflict of interest.

## Abbreviations

MPM: Multi-sequence method
SPM: Single-sequence method
IBPMP: Improved Base Pair Maximization Principle
CSCP: Candidate stems combination principle
PCR: Prediction complementary region.
