# Supplementary material for: DMfold: A Novel Method to Predict RNA Secondary Structure With Pseudoknots Based on Deep Learning and Improved Base Pair Maximization Principle
Source: Front Genet. 2019 Mar 4;10:143. doi: 10.3389/fgene.2019.00143 (PMC6409321; doi:10.3389/fgene.2019.00143)
Supplement: Supplementary file 1 [file Data_Sheet_1.ZIP › Supplementary/Supplementary_Material.docx]

Supplementary Material

# Supplementary Data

## The detail of one-hot encoding in RNA sequences

RNA sequences contain four possible bases: adenine(A), guanine(G), cytosine(C), or uracil(U). Under normal physiological conditions, these bases can bind with one another through the hydrogen-bond to form base pairs. Hence, the one-hot code of each base contains two parts. The first part is the one-hot code of bases, such as: A is 1000, U is 0010, G is 0100, C is 0010 and the padding base N is 0000. The secondary part is the one-hot code of match base based on the Watson-Crick pairs, such as A is 0010, U is 1000, G is 0010, C is 0100 and the padding base N is 0000. Therefore, the code of A is 10000010, U is 00101000, G is 01000010, C is 00100100 and N is 00000000.

## The detail of one-hot encoding in dot-bracket sequences

Dot-bracket sequences contain seven symbols. Hence, the one-hot code of each symbol is the final code, such as: ‘(’ is 1000000, ‘)’ is 0000001, ‘.’ is 0001000, ‘[’ is 0100000, ‘]’ is 0000010, ‘{’ is 0010000, ‘}’ is 0000100 and the padding symbol ‘N’ is 0000000.

## Structure Visualization Comparison

In this section, the other three families (5sRNA, tmRNA and RNaseP) of RNA are also performed structure visualization comparison. One RNA molecule is randomly selected from each of the other three families of the test set, and the prediction results of those six methods are obtained. Then use the forna tool to get the visualization maps of those prediction results. **Figure S4** shows the visual representation of the real and prediction structures of 5sRNA (5s_Andrias-japonicus-2,). **Figure S5** shows the visual representation of the real and prediction structures of tmRNA (tmRNA_Bifi.long._TRW-206672_1-397). **Figure S6** shows the visual representation of the real and prediction structures of RNaseP (RNaseP_A.ferrooxidans).

It can be found in **Figure S4**, the shape of our method is the closest to the natural structure, and it contains almost all stems and loops in the natural structure. Although there are some differences between our structure and the real structure in **Figure S5**, the shape of our method is still the closest to the natural structure, and our method successfully predicts two pseudoknots. Other structures, although they also contain pseudoknots, those pseudoknots are not correct. In **Figure S6**, our structure is still the closest to the natural structure, and correctly predict a pseudoknot. In all three figures, the prediction results of our method are the closest to the natural structure. Hence, our method performs better than other methods.

# 2 Supplementary Figures and Tables

Table S1: The detail of Original-Data, Experimental-Data and Cleared-Data.
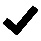
 represents the set contains corresponding family RNA data. On the contrast,
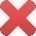
 represents the set does not contain corresponding family RNA data. The ‘Number’ is used to record the number of RNA in a set.

| **RNA Family** | **Original_Data** | | **Experimental_Data** | | **Clean_Data** | |
| --- | --- | --- | --- | --- | --- | --- |
|  | **Family** | **Number** | **Family** | **Number** | **Family** | **Number** |
| tmRNA | 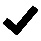 | 462 | 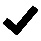 | 462 | 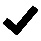 | 348 |
| tRNA | 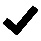 | 557 | 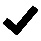 | 557 | 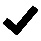 | 432 |
| 5sRNA | 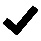 | 1283 | 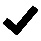 | 1283 | 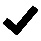 | 946 |
| 16sRNA | 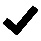 | 110 | 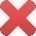 | 0 | 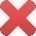 | 0 |
| 23sRNA | 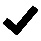 | 35 | 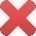 | 0 | 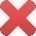 | 0 |
| RNasep | 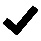 | 454 | 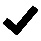 | 454 | 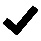 | 383 |
| Telomerase_RNA | 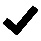 | 37 | 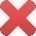 | 0 | 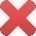 | 0 |
| Srp_RNA | 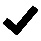 | 928 | 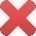 | 0 | 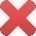 | 0 |
| Grp_RNA | 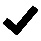 | 109 | 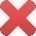 | 0 | 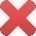 | 0 |

**Figure S1.** LSTM memory cell with gating units.

**Figure S2.** The schematic diagram of the encoder, which contains three layers Bi-LSTM. Each layer contains two initial vectors with dimension 1*300. The input is the one-hot vector (1*8) of each base and the output for each base is a vector with dimension 1*600.

**Figure S3.** The schematic diagram of the decoder, which contains four layers nodes.

**Figure S4.** The structure visualization comparison of 5sRNA. Green bases represent the stem. Red bases represent the bifurcation loop and unpaired single chain. Blue bases represent the hairpin loop. Yellow bases represent the interior and bulge loop. Gray lines represent pseudoknot.

**Figure S5.** The structure visualization comparison of tmRNA. Green bases represent the stem. Red bases represent the bifurcation loop and unpaired single chain. Blue bases represent the hairpin loop. Yellow bases represent the interior and bulge loop. Gray lines represent pseudoknot.

**Figure S6.** The structure visualization comparison of RNaseP. Green bases represent the stem. Red bases represent the bifurcation loop and unpaired single chain. Blue bases represent the hairpin loop. Yellow bases represent the interior and bulge loop. Gray lines represent pseudoknot.
