# Supplementary figures and images for: DMfold: A Novel Method to Predict RNA Secondary Structure With Pseudoknots Based on Deep Learning and Improved Base Pair Maximization Principle
Source: Front Genet. 2019 Mar 4;10:143. doi: 10.3389/fgene.2019.00143 (PMC6409321; doi:10.3389/fgene.2019.00143)

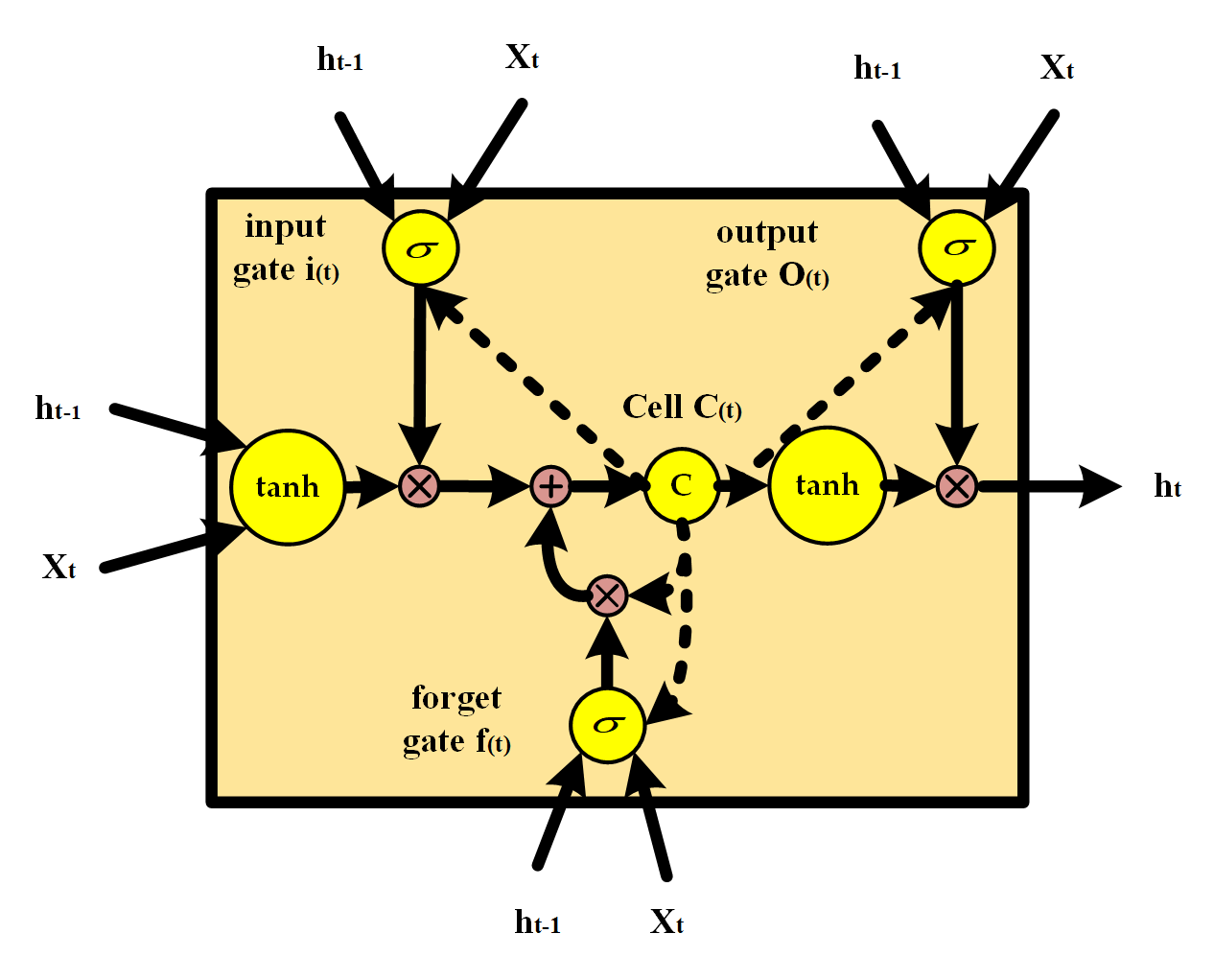

Supplement: Supplementary file 1 [file Data_Sheet_1.ZIP › Supplementary/Figure S1.tif]

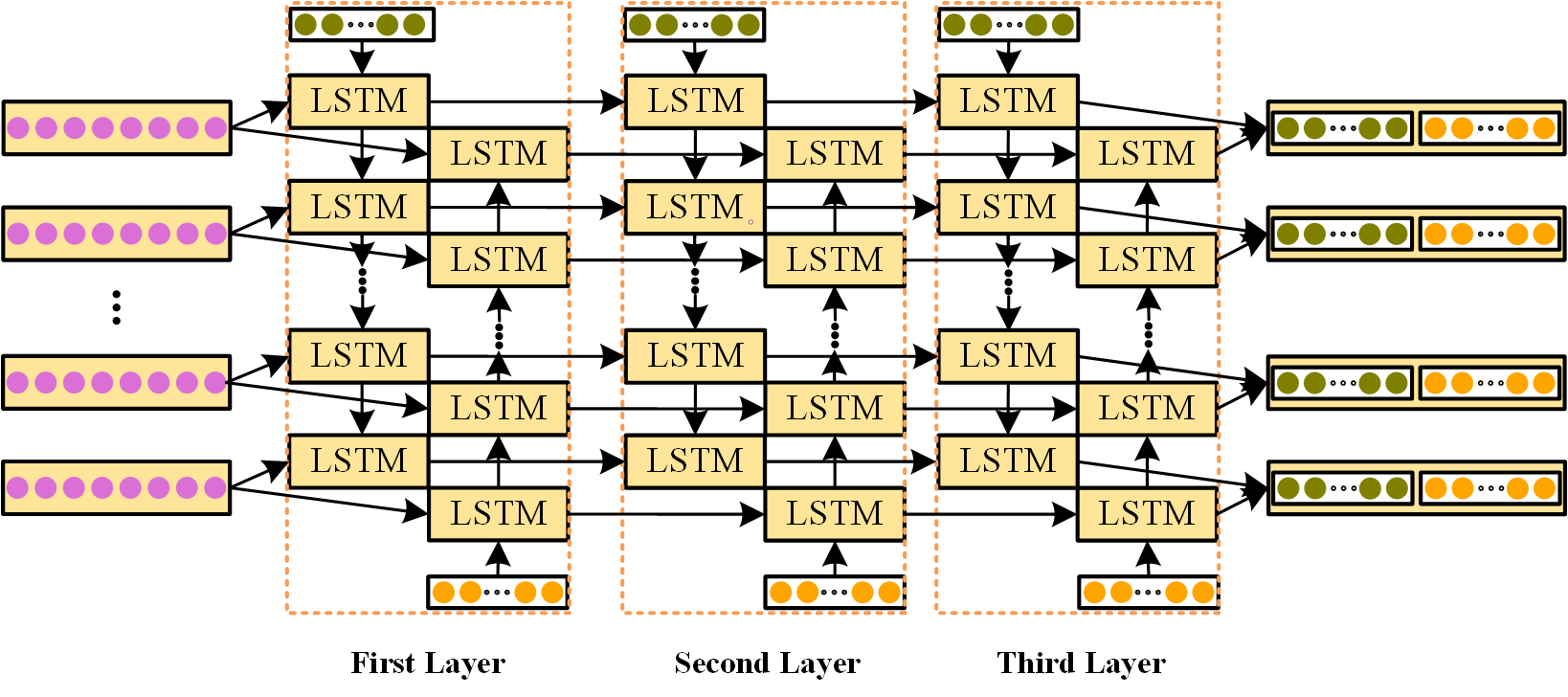

Supplement: Supplementary file 1 [file Data_Sheet_1.ZIP › Supplementary/Figure S2.TIF]

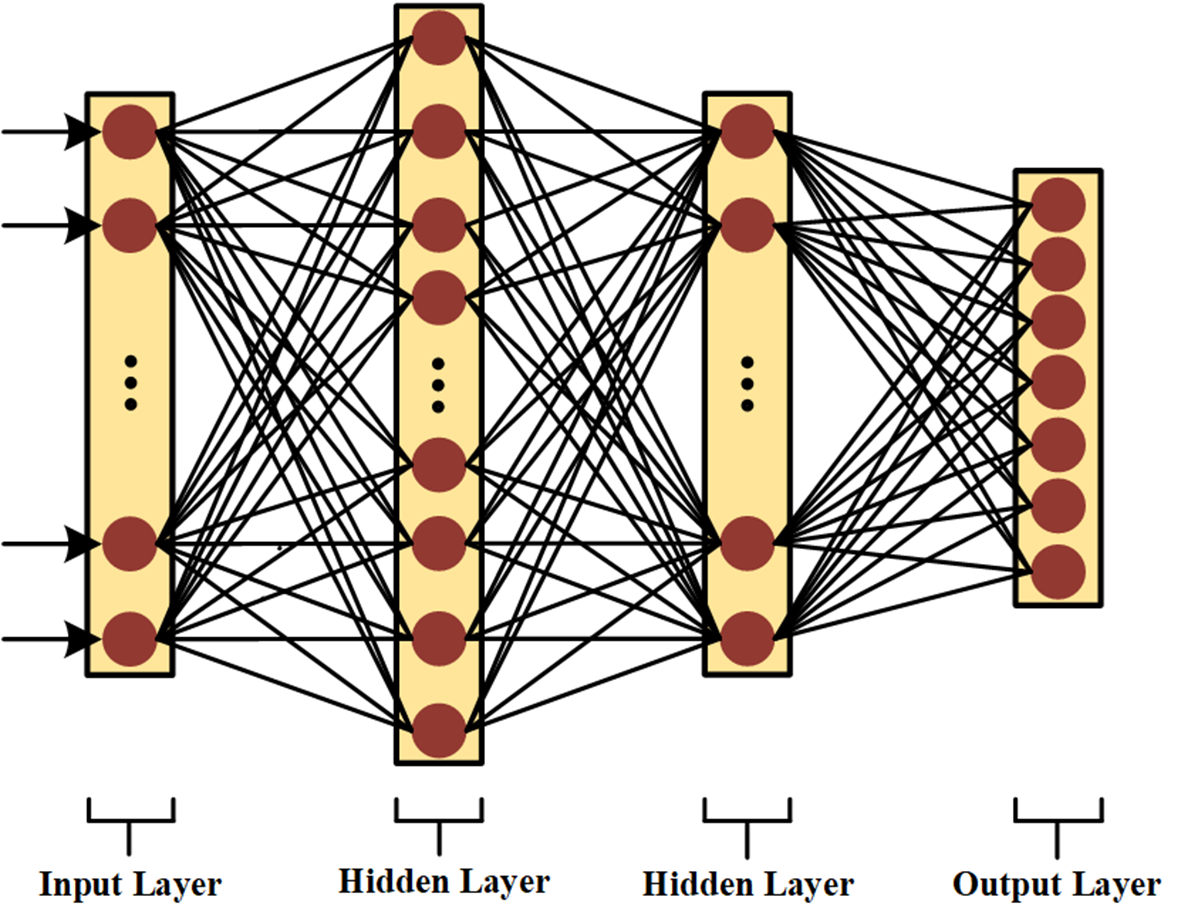

Supplement: Supplementary file 1 [file Data_Sheet_1.ZIP › Supplementary/Figure S3.TIF]
